# Subtype C ALVAC-HIV and bivalent subtype C gp120/MF59 HIV-1 vaccine in low-risk, HIV-uninfected, South African adults: a phase 1/2 trial

**DOI:** 10.1016/S2352-3018(18)30071-7

**Published:** 2018-06-18

**Authors:** Linda-Gail Bekker, Zoe Moodie, Nicole Grunenberg, Fatima Laher, Georgia D Tomaras, Kristen W Cohen, Mary Allen, Mookho Malahleha, Kathryn Mngadi, Brodie Daniels, Craig Innes, Carter Bentley, Nicole Frahm, Daryl E Morris, Lynn Morris, Nonhlanhla N Mkhize, David C Montefiori, Marcella Sarzotti-Kelsoe, Shannon Grant, Chenchen Yu, Vijay L Mehra, Michael N Pensiero, Sanjay Phogat, Carlos A DiazGranados, Susan W Barnett, Niranjan Kanesa-thasan, Marguerite Koutsoukos, Nelson L Michael, Merlin L Robb, James G Kublin, Peter B Gilbert, Lawrence Corey, Glenda E Gray, M Juliana McElrath, HVTN 100 Protocol Team

**Affiliations:** a The Desmond Tutu HIV Centre, University of Cape Town, Cape Town, South Africa; b Vaccine and Infectious Disease Division, Fred Hutchinson Cancer Research Center, Seattle, WA, USA; c Perinatal HIV Research Unit, Faculty of Health Sciences, University of the Witwatersrand, Johannesburg, South Africa; d Duke Human Vaccine Institute, Duke University School of Medicine, Durham, NC, USA; e Vaccine Research Program, Division of AIDS, National Institute of Allergy and Infectious Diseases, National Institutes of Health, Bethesda, MD, USA; f Setshaba Research Centre, Soshanguve, Pretoria, South Africa; g Centre for the Programme of Aids Research in South Africa (CAPRISA), Durban, South Africa; h School of Laboratory Medicine and Medical Sciences, University of KwaZulu-Natal, Durban, South Africa; i South African Medical Research Council, Durban, South Africa; j The Aurum Institute, Klerksdorp Research Centre, Klerksdorp, South Africa; k National Institute for Communicable Diseases, National Health Laboratory Service, Johannesburg, South Africa; l Sanofi Pasteur, Swiftwater, PA, USA; m GSK Vaccines, Cambridge, MA, USA; n GSK Vaccines, Rockville, MD, USA; o GSK Vaccines, Rixensart, Belgium; p US Military HIV Research Program, Walter Reed Army Institute of Research, Silver Spring, MD, USA; q South African Medical Research Council, Cape Town, South Africa; r Bill & Melinda Gates Foundation, Seattle, WA, USA; s Kanesa LLC, Lexington, MA, USA

## Abstract

**Background:**

Modest efficacy was reported for the HIV vaccine tested in the RV144 trial, which comprised a canarypox vector (ALVAC) and envelope (env) glycoprotein (gp120). These vaccine components were adapted to express HIV-1 antigens from strains circulating in South Africa, and the adjuvant was changed to increase immunogenicity. Furthermore, 12-month immunisation was added to improve durability. In the HIV Vaccine Trials Network (HVTN) 100 trial, we aimed to assess this new regionally adapted regimen for advancement to efficacy testing.

**Methods:**

HVTN 100 is a phase 1/2, randomised controlled, double-blind trial at six community research sites in South Africa. We randomly allocated adults (aged 18–40 years) without HIV infection and at low risk of HIV infection to either the vaccine regimen (intramuscular injection of ALVAC-HIV vector [vCP2438] at 0, 1, 3, 6, and 12 months plus bivalent subtype C gp120 and MF59 adjuvant at 3, 6, and 12 months) or placebo, in a 5:1 ratio. Randomisation was done by computer-generated list. Participants, investigators, and those assessing outcomes were masked to random assignments. Primary outcomes included safety and immune responses associated with correlates of HIV risk in RV144, 2 weeks after vaccination at 6 months (month 6·5). We compared per-protocol participants (ie, those who completed the first four vaccinations and provided samples at month 6·5) from HVTN 100 with stored RV144 samples assayed contemporaneously. This trial is registered with the South African National Clinical Trials Registry (DOH-27-0215-4796) and ClinicalTrials.gov (NCT02404311).

**Findings:**

Between Feb 9, 2015, and May 26, 2015, 252 participants were enrolled, of whom 210 were assigned vaccine and 42 placebo. 222 participants were included in the per-protocol analysis (185 vaccine and 37 placebo). 185 (100%) vaccine recipients developed IgG binding antibodies to all three vaccine-matched gp120 antigens with significantly higher titres (3·6–8·8 fold; all p<0·0001) than the corresponding vaccine-matched responses of RV144. The CD4+ T-cell response to the ZM96.C env protein in HVTN 100 was 56·4% (n=102 responders), compared with a response of 41·4% (n=79 responders) to 92TH023.AE in RV144 (p=0·0050). The IgG response to the 1086.C variable loops 1 and 2 (V1V2) env antigen in HVTN 100 was 70·5% (95% CI 63·5–76·6; n=129 responders), lower than the response to V1V2 in RV144 (99·0%, 95% CI 96·4–99·7; n=199 responders).

**Interpretation:**

Although the IgG response to the HVTN 100 vaccine was lower than that reported in RV144, it exceeded the predicted 63% threshold needed for 50% vaccine efficacy using a V1V2 correlate of protection model. Thus, the subtype C HIV vaccine regimen qualified for phase 2b/3 efficacy testing, a critical next step of vaccine development.

**Funding:**

US National Institute of Allergy and Infectious Diseases (NIAID), and Bill & Melinda Gates Foundation.

## Introduction

Of six preventive HIV-1 vaccine efficacy trials done to date,^1^, ^2^, ^3^, ^4^, ^5^, ^6^ only the RV144 trial has provided any indication that vaccination can prevent HIV acquisition.^5^ RV144 was done with more than 16 000 participants aged 18–30 years in Thailand, where HIV subtype CRF01_AE is prevalent.^7^ The vaccine regimen was two doses of the replication-defective canarypox-HIV recombinant ALVAC-HIV vector (vCP1521) followed by two doses of vCP1521 plus alum-adjuvanted AIDSVAX subtypes B/E HIV envelope (env) glycoprotein (gp120). The observed vaccine efficacy over the first 3·5 years was 31·2% (95% CI 1·1–52·1; p=0·04).^5^ Mathematical modelling has indicated that the HIV pandemic could be slowed markedly by a regimen with 50% vaccine efficacy.^8^ A post-hoc analysis of RV144 data showed that vaccine efficacy exceeded this benchmark over the first year (vaccine efficacy 60·5%, 95% CI 22–80),^9^ suggesting that improving durability of immune responses induced by the RV144 vaccine regimen could have a substantial effect.

**Research in context**

**Evidence before this study**

We searched PubMed up to the end of March, 2018, with the terms “HIV vaccine efficacy trial”, “RV144”, “ALVAC”, and “HIV vaccine development”. We did not restrict our search by language. Dozens of candidate HIV vaccines have entered clinical testing; initially, HIV envelope (env) glycoproteins were identified as potential targets for neutralising antibodies, and various HIV-1 env immunogens were proposed to elicit such antibodies. Clinical testing between 1986 and 2003 culminated in negative findings in the first two efficacy trials of gp120 env immunogens—VAX003 in Thailand and VAX004 in North America. Subsequently, focus shifted towards cytotoxic T lymphocytes as a potential mechanism of protection and development of poxvirus and adenovirus vector vaccines to elicit cytotoxic-T-lymphocyte responses. Poxvirus vector vaccines had been in development since the mid-1990s, but the first such vaccine to advance to efficacy testing was an adenovirus serotype 5 (Ad5) vector vaccine developed by Merck. An interim review in 2007 found that vaccination with the Merck Ad5 vector vaccine seemed to increase the risk of infection; therefore, that trial (Step) and its South African sister trial (Phambili) were both stopped. During this same period, however, the US Military HIV Research Program and the Thai Ministry of Health were undertaking the RV144 trial, which tested the efficacy of a prime-boost regimen containing canarypox vector (ALVAC) and gp120 env vaccines. In 2009, results from this trial were announced, and a modest reduction in risk of HIV infection was recorded in the vaccine group compared with the placebo group. Based on these encouraging results, in 2010, international funders and scientific partners in HIV vaccine research met to build on the RV144 results to address the HIV epidemic in sub-Saharan Africa. Subsequently, a systematic analysis of potential vaccine strains resulted in an HIV-1 subtype C-based prime-boost vaccine regimen using the ALVAC vector backbone (as in RV144) with clade B and C HIV-1 gene inserts and bivalent subtype C recombinant HIV env gp120. The squalene-based emulsion MF59 was selected as an adjuvant with the goal of increasing immunogenicity and improving on the efficacy reported in RV144. The HIV Vaccine Trials Network (HVTN) 100 trial was designed to assess the safety and immunogenicity of this vaccine regimen, particularly in the context of vaccine-induced immune responses that correlated with reduced risk of HIV infection in RV144.

**Added value of the study**

On the basis of immune correlates of HIV-1 infection risk identified in the RV144 trial, criteria were selected to qualify the HVTN 100 regimen for efficacy testing. These criteria were selected such that extrapolation of the RV144 results would project to meet targeted reductions in HIV infection risk in a subsequent efficacy trial. This report is the first description of the vaccine regimen and the immunogenicity tests done to determine whether to advance this regimen to pivotal efficacy testing.

**Implications of all the available evidence:**

The subtype C vaccine regimen induced strong humoral and cellular responses and met prespecified criteria supporting assessment for preventive efficacy. This vaccine regimen is now under evaluation in the HVTN 702 phase 2b/3 efficacy trial in South Africa. Additional innovative HIV vaccine strategies continue in development (eg, alternative adenovirus vectors with mosaic inserts, sequential vaccination strategies to elicit known broadly neutralising antibodies against HIV-1, replication-competent viral vectors, polyvalent env glycoproteins, alternative vaccine adjuvants). However, the HVTN 100 vaccine regimen is the first designed specifically to extend and improve on a vaccine regimen that has shown efficacy—albeit modest—in reducing the risk of HIV infection.

To define immune responses associated with vaccine efficacy, case-control immune correlate analyses were done on samples from the peak immunogenicity timepoint in RV144 (month 6·5, 2 weeks after the fourth vaccination).^10^ The initial assessment identified two primary immune correlates of risk of HIV acquisition. First, an inverse correlation with infection rate was noted with the presence of IgG antibody that bound to a gp70-scaffolded HIV-1 env variable loops 1 and 2 (V1V2) recombinant protein (CaseA2_gp70_V1V2.B).^11^ Second, a direct correlation with infection rate was recorded with plasma env-specific binding IgA. Four other primary variables—antibody-dependent cellular toxicity, IgG antibody avidity, neutralising antibodies, and env-specific CD4+ T cells—correlated inversely with infection rate only when IgA binding was low. Secondary analyses showed additional correlates of risk, including binding IgG antibodies to vaccine-matched gD-gp120 proteins A244.AE and 92TH023.AE.^10^ Subsequent analyses identified functionality and polyfunctionality scores of env-specific CD4+ T-cell responses as independent correlates of risk^12^ and indicated that V1V2 IgG3 responses correlated with decreased risk of HIV-1 infection.^13^, ^14^ The importance of V2 antibody responses in vaccine-mediated protection was substantiated by viral sieve analyses.^15^, ^16^

To build on (and potentially enhance) the RV144 trial results, we adapted the Thai vaccine regimen for the sub-Saharan African region, where HIV subtype C is prevalent, the burden of HIV disease is greatest, and a vaccine against HIV is needed most urgently. We aimed to improve vaccine efficacy by increasing the magnitude and duration of vaccine-elicited immune responses beyond those of RV144. The new regionally adapted vaccine regimen maintained the basic canarypox vector prime (ALVAC) and recombinant gp120 boost utilised in the RV144 regimen while incorporating clade C immunogens and substituting the alum adjuvant used in RV144 for MF59. The new vaccination schedule added an immunisation boost at month 12 (the fifth vaccination).

The HIV Vaccine Trials Network (HVTN) 100 trial is a first-in-human trial of this regionally adapted vaccine regimen. We aimed to assess the safety and immunogenicity of the new vaccine in adults living in South Africa at the primary, prespecified, peak immunogenicity timepoint 2 weeks after the fourth vaccination (month 6·5). We compared HVTN 100 peak immunogenicity with contemporaneously assayed, archived blood samples from a new random sample of HIV-uninfected RV144 participants. Based on the correlates of risk described earlier, we used four prespecified immunological criteria associated with vaccine take, potency, and correlates of risk in RV144 to guide the decision of whether to proceed to a phase 2b/3 efficacy trial. Here we report the primary immunogenicity results and supportive peak immunogenicity analyses. Blinded long-term follow-up is ongoing in HVTN 100 to assess further safety, longer term durability, and responses induced after an additional boost in a study extension. To preserve masking, we do not present safety data here. The National Institute of Allergy and Infectious Diseases (NIAID) Data and Safety Monitoring Board (DSMB) does semi-annual reviews of HVTN 100 data and no safety concerns have been identified to date.

## Methods

### Participants

We did a randomised, controlled, double-blind study at six community research sites in South Africa: Cape Town (Western Cape), eThekwini and Isipingo (KwaZulu-Natal), Klerksdorp (Northwest province), and Soweto and Soshanguve (Gauteng). The Cape Town, eThekwini, and Soweto sites are affiliated with academic hospitals.

Volunteers were eligible for enrolment if they were aged 18–40 years, could give written informed consent, were healthy, were not infected with HIV, were at low risk for HIV acquisition, and had not previously received an HIV vaccine. We defined low risk for HIV acquisition as either being sexually abstinent, in a mutually monogamous relationship with a partner known to have HIV-uninfected status, or having one partner believed not to be infected with HIV and with whom he or she regularly used condoms for vaginal or anal intercourse; furthermore, participants had to have no history of newly acquired sexually transmitted infections in the 12 months before enrolment. We required women to be on contraception, not pregnant, and non-lactating. To achieve a relative balance of sexes, we monitored enrolment to ensure no more than 60% of trial participants of either sex were enrolled.

The research ethics committees of the University of the Witwatersrand, the University of Cape Town, the University of KwaZulu-Natal, and the Medical Research Council approved the study. All participants gave written informed consent in English or their local language (Setswana, Sotho, Xhosa, or Zulu).

### Randomisation and masking

We randomly assigned participants to receive vaccine or placebo in a 5:1 ratio. The statistical centre (Seattle, WA, USA) produced the block-randomised sequence by computer-generated random numbers, which were provided to every study site through a web-based randomisation system. Participants, site staff who enrolled and followed up participants, the study team (except biostatisticians), and laboratory personnel were masked to participant group assignments. Site pharmacists were aware of the random assignment to ensure proper study product handling and dispensing, which included application of overlays to all syringes for masking before delivery to site staff. NIAID Division of AIDS (DAIDS) protocol pharmacists, contract monitors, and data management centre staff, and the NIAID DSMB, were unmasked to ensure proper trial conduct and safety review. The trial remains masked at the participant level because a protocol extension is ongoing.

### Procedures

The investigational products were ALVAC-HIV (vCP2438), which was manufactured by IDT Biologika (Dessau-Rosslau, Germany) for Sanofi Pasteur, and bivalent subtype C gp120, which was manufactured by Rentschler Biotechnologie (Laupheim, Germany) for Novartis Vaccines and Diagnostics (now GlaxoSmithKline Vaccines).

ALVAC-HIV (vCP2438) is a preparation of live attenuated recombinant canarypox-derived virus expressing products from the HIV-1 *env* gp120 (subtype C ZM96 [based on HIV-1 96ZM651]), the transmembrane region of *env* gp41, *gag*, and *protease* (all subtype B HIV-1 LAI) coding sequences, and cultured in primary chicken embryo fibroblasts. The recombinant canarypox backbone vector used in ALVAC-HIV (vCP2438) was the same as that used for vCP1521 in the RV144 vaccine regimen, but the CRF01_AE gp120 insert (92TH023) was exchanged for a subtype C gp120 insert (96ZM651). The vector was formulated as a lyophilised vaccine reconstituted in sterile sodium chloride solution (NaCl 0·4%) for intramuscular injection as one dose (viral titre nominal dose of 10^7^ 50% cell culture infectious dose [CCID50]) at each vaccination. The placebo for ALVAC-HIV was a mixture of virus stabiliser and freeze-drying medium reconstituted with 0·4% NaCl.

Bivalent subtype C gp120 consisted of two subtype C recombinant monomeric env proteins, TV1.C and 1086.C gp120s,^17^ which replaced the bivalent A244 (CRF01_AE) and subtype B MN gp120 proteins used in RV144. This vaccine component was delivered as a 0·5 mL intramuscular injection, consisting of 100 μg of each recombinant protein (which is a third of the 300 μg per protein alum-adjuvanted dose given in RV144) combined with the MF59 adjuvant—a protein dose-sparing squalene oil-in-water emulsion.^18^, ^19^, ^20^ The placebo for bivalent subtype C gp120/MF59 was 0·9% NaCl.

At screening for the study, we obtained the participant's consent and assessed their understanding of the study. We then took a medical history, did a complete physical examination and behavioural risk assessment, and did procedures including a screening rapid HIV test, urine dipstick, and pregnancy test. We also collected blood samples for complete blood count, chemistry panel, and testing for hepatitis B and C and syphilis.

Participants assigned placebo received injections at months 0, 1, 3, 6, and 12. Participants assigned vaccine received ALVAC-HIV (vCP2438) at months 0 and 1 followed by ALVAC-HIV (vCP2438) plus bivalent subtype C gp120/MF59 at months 3, 6, and 12. Primary immunogenicity endpoints are based on data at month 6·5 (ie, 2 weeks after fourth dose).

We gathered safety data including local and systemic reactogenicity signs and symptoms occurring within 3 days after every vaccination, unsolicited adverse events occurring within 30 days after every vaccination, serious adverse events occurring throughout the duration of the trial, and all adverse events leading to early participant withdrawal or early discontinuation of study product administration. We graded adverse events according to the DAIDS Table for Grading the Severity of Adult and Pediatric Adverse Events, version 2.0. The Protocol Safety Review Team monitored masked safety reports routinely, and the DSMB reviewed unblinded safety reports biannually.

Participants returned to the study site for safety follow-up visits 2 weeks after every vaccination and for in-study HIV diagnostic testing every 3 months. At all visits, we did clinical assessments and risk reduction counselling. We followed up all participants for 18 months from enrolment. We recruited a subset of participants to receive a booster vaccination at month 30, with follow-up until month 36. Follow-up is ongoing and safety data remain blinded for all HVTN 100 participants until 6 months after the last booster vaccination.

All assays were done in HVTN laboratories by staff who were unaware of treatment assignments, and validated methods were used.^10^, ^21^, ^22^, ^23^

CD4+ T-cell responses to HIV vaccine insert-matched peptides were measured by intracellular cytokine staining. The assay detects the production and accumulation of cytokines on inhibition of intracellular transport after brief cell stimulation. Cryopreserved peripheral blood mononuclear cells were thawed, rested overnight, and stimulated with 11 aminoacid-overlapping 15-mer peptide pools representing ALVAC inserts ZM96 gp120 (for HVTN 100) and 92TH023 gp120 (for RV144),^22^, ^24^ dimethyl sulfoxide (DMSO; negative control) or *staphylococcal enterotoxin B* (positive control) in the presence of costimulatory antibodies (CD28 and CD49d), and intracellular transport inhibitors brefeldin A and monensin for 6 h at 37°C. Next, cells were washed and incubated with EDTA (edetic acid) overnight at 4°C, then stained with a 16-colour panel,^24^ acquired on a BD LSRII flow cytometer (BD Biosciences, San Jose, CA, USA), and analysed using FlowJo version 9.9.4 (BD, Franklin Lakes, NJ, USA). Data were excluded from subsequent analyses if background responses (DMSO control) were greater than 0·1% cytokine-positive, or if fewer than 5000 CD4+ T cells were acquired. Positive response criteria are in the appendix (p 2).

HVTN 100 serum and RV144 plasma HIV-1-specific IgG binding antibody responses were measured at dilutions of 1:40 (IgG3 to gp120 and V1V2 antigens), 1:100 (IgG to V1V2 antigens), or 1:200 (IgG to gp120 antigens) by an HIV-1 binding antibody multiplex assay.^10^, ^23^ Antigen and positive response criteria descriptions are in the appendix (pp 2, 7). To account for differences between serum and plasma in these assays, we did a sensitivity analysis to assess whether any differences in responses and magnitudes among positive responders to gp120 and V1V2 antigens in HVTN 100 are still seen after applying a mean location shift of −0·10 log_10_ to HVTN 100 responses. We also measured IgG3 responses to gp120 and V1V2 in the HVTN 100 and RV144 cohorts, because IgG3 binding antibody responses seemed to differentiate the RV144 regimen containing ALVAC priming and gp120 boosting from the ineffective gp120 alone regimen tested in VAX003 and were associated with decreased HIV-1 risk.^14^, ^25^

Neutralising anwtibodies against HIV-1 were measured as a function of reductions in Tat-regulated luciferase (Luc) reporter gene expression in TZM-bl cells. The assay measured neutralisation titres against a panel of heterologous HIV-1 subtype C env-pseudotyped viruses with neutralisation phenotypes ranging from very high sensitivity to antibody-mediated neutralisation (ie, Tier 1A;^26^ MW965.26.C, CH0505.w4.3.C, and SO032_A2.8-1.C) to above-average sensitivity to antibody-mediated neutralisation (ie, Tier 1B;^26^ 6644.V2.C33.C, CA146 H3.3.C, and 1107356.07.C). Titres against autologous env-pseudotyped viruses from the vaccine strains (96ZM651.C and Ce1086_B2.C [both Tier 2—ie, moderate sensitivity to antibody-mediated neutralisation]^26^ and TV1c8.2.C [Tier 1A]) were also assessed together with TV1.21.C, a related virus with a Tier 2 phenotype. Titre was defined as the serum dilution that reduced relative luminescence units by 50% compared with relative luminescence units in virus control wells (cells and virus only) after subtraction of background relative luminescence units (cells only). If a titre was left-censored, half the left limit was used as the titre value. A response was judged positive if the neutralisation titre was above ten (half the lowest dilution tested).

### Outcomes

The primary objectives of the study were to evaluate the safety, tolerability, and immunogenicity of the vaccine regimen after two doses of ALVAC-HIV (vCP2438) followed by two doses of ALVAC-HIV (vCP2438) and bivalent subtype C gp120/MF59 (2 weeks after the fourth vaccination, month 6·5). The primary safety endpoints included local and systemic reactogenicity signs and symptoms through the first four vaccinations and unsolicited adverse events. Primary immunogenicity endpoints of the prespecified immunological criteria are described in the appendix (p 2).

### Statistical analysis

To compare immune response data from HVTN 100 with that from RV144, we drew a pilot sample from RV144 of vaccine and placebo recipients who were not infected with HIV, based on stratified random sampling by sex and number of vaccinations received to match the strata ratios in HVTN 100. We selected the sample sizes of HVTN 100 (n=210 vaccine, n=42 placebo) and the subset of RV144 (n=212 vaccine, n=24 placebo) to provide 90% power to show the HVTN 100 regimen had potential for further clinical development.

We based immunogenicity analyses on the per-protocol cohorts of HVTN 100 and the RV144 subset, which consisted of all participants who received the first four scheduled vaccinations. We excluded participants who had an HIV-1 positive test by month 6·5.

Boxplots show distributions of immune responses to individual antigens at the peak immunogenicity timepoint. We summarised immune responses by the proportion of participants with a positive response and by magnitude (geometric mean titre [GMT] for humoral assays, % T cells expressing marker combination for cellular assays). We calculated two-sided 95% CIs for positive response rates (or difference in these) using the Wilson (Miettinen-Nurminen) method. We plotted the magnitude–breadth curve and calculated the area under the curve (AUC) as a summary measure,^27^ to describe the magnitude and breadth across a panel of antigens. We analysed antigen-specific T-cell subsets by COMPASS (combinatorial polyfunctionality analysis of antigen-specific T-cell subsets),^12^ and we defined the functionality score as the estimated proportion of env-specific subsets detected among all possible subsets. The polyfunctionality score is similar but weights the different subsets by their degree of functionality, favouring subsets with higher degrees of functions.^12^ We reported COMPASS posterior probabilities and correlations between pairs of immune responses for HVTN 100 and RV144. We did principal components analysis biplots to show multivariate immune response data in HVTN 100 and RV144.

We used Fisher's exact test and Wilcoxon rank-sum test to compare immune response rates and magnitudes among positive responders, respectively, between the two independent vaccine groups in HVTN 100 and RV144. We used Wilcoxon rank-sum test to compare AUCs between the HVTN 100 and RV144 vaccine groups. All p values are two-sided; we judged a p value less than 0·05 significant. We did statistical analyses using SAS (version 9.4; SAS Institute, Cary, NC, USA) and R statistical software (version 2.15.1; R Foundation for Statistical Computing, Vienna, Austria).

The NIAID DSMB provided study oversight. The trial was registered with the South African National Clinical Trials Registry (DOH-27-0215-4796) and ClinicalTrials.gov (NCT02404311).

### Data sharing

The data and protocol are available online.

### Role of the funding source

The funders contributed to, reviewed, and approved the HVTN 100 study design, established the prespecified immunological criteria for advancing this regimen, and reviewed data from HVTN 100 against those criteria. The corresponding author had full access to all data in the study and had final responsibility for the decision to submit for publication.

## Results

**Figure 1:**
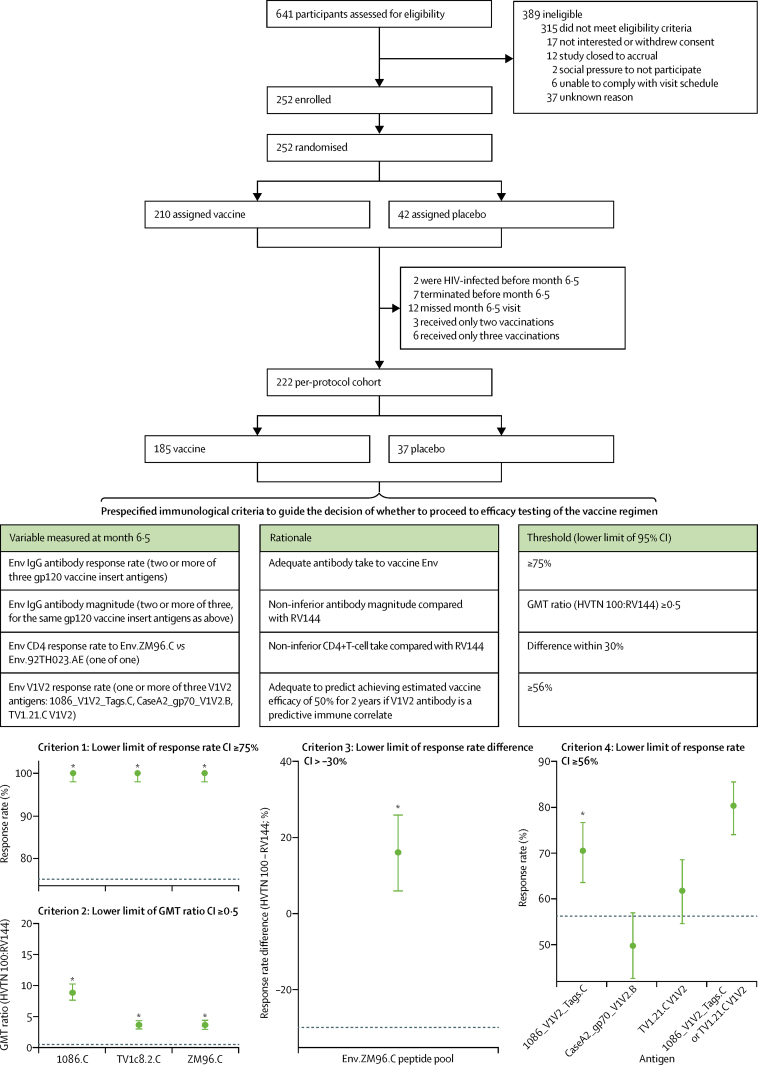
HVTN 100 trial profile and prespecified immunological criteria scoring Env=envelope. GMT=geometric mean titre. V1V2=variable loops 1 and 2. *Prespecified immunological criterion passed.

Between Feb 9, 2015, and May 26, 2015, 252 participants were enrolled in HVTN 100, of whom 210 were allocated vaccine and 42 placebo (figure 1). 109 (43%) participants were women and 246 (98%) were black (table 1). Median age was 23 years (IQR 21–27). Baseline characteristics were balanced between the vaccine and placebo groups. Of those enrolled, 222 were in the per-protocol cohort (185 assigned vaccine and 37 placebo), which comprised people who received the first four vaccinations as scheduled and provided samples at the month 6·5 visit (figure 1). Two participants became infected with HIV before month 6·5 and were not included in the analysis. Masked long-term follow-up is ongoing.

**Table 1:** Baseline characteristics of the intention-to-treat and per-protocol cohorts of HVTN 100 and RV144

	**Intention-to-treat cohort**		Per-protocol cohort*	
	HVTN 100 (n=252)	RV144 (n=236)†	HVTN 100 (n=222)	RV144 (n=225)
**Treatment**				
Placebo	42 (17%)	24 (10%)	37 (17%)	24 (11%)
Vaccine	210 (83%)	212 (90%)	185 (83%)	201 (89%)
**Age group (years)**				
18–20	56 (22%)	66 (28%)	52 (23%)	60 (27%)
21–25	107 (42%)	112 (47%)	96 (43%)	111 (49%)
≥26	89 (35%)	58 (25%)	74 (33%)	54 (24%)
**Sex**‡				
Female	109 (43%)	98 (42%)	91 (41%)	91 (40%)
Male	143 (57%)	138 (58%)	131 (59%)	134 (60%)
**Body-mass index (kg/m^2^)**§				
0–25	162 (66%)	NA	145 (67%)	NA
25–30	52 (21%)	NA	46 (21%)	NA
≥31	30 (12%)	NA	24 (11%)	NA

Data are number of participants (%). NA=not available.

* Per-protocol HVTN 100 and RV144 cohorts include participants who received the first four scheduled vaccinations and did not have HIV infection at month 6·5; two participants in HVTN 100 were infected with HIV before month 6·5.

† RV144 cohort selected from participants in RV144 not infected with HIV, frequency-matched to HVTN 100 participants by sex and number of vaccinations.

‡ Sex options in HVTN 100 included trans and self-identify; one HVTN 100 participant self-identified as homosexual male; all other participants reported male or female.

§ Body-mass index was not measured in RV144.

**Figure 2:**
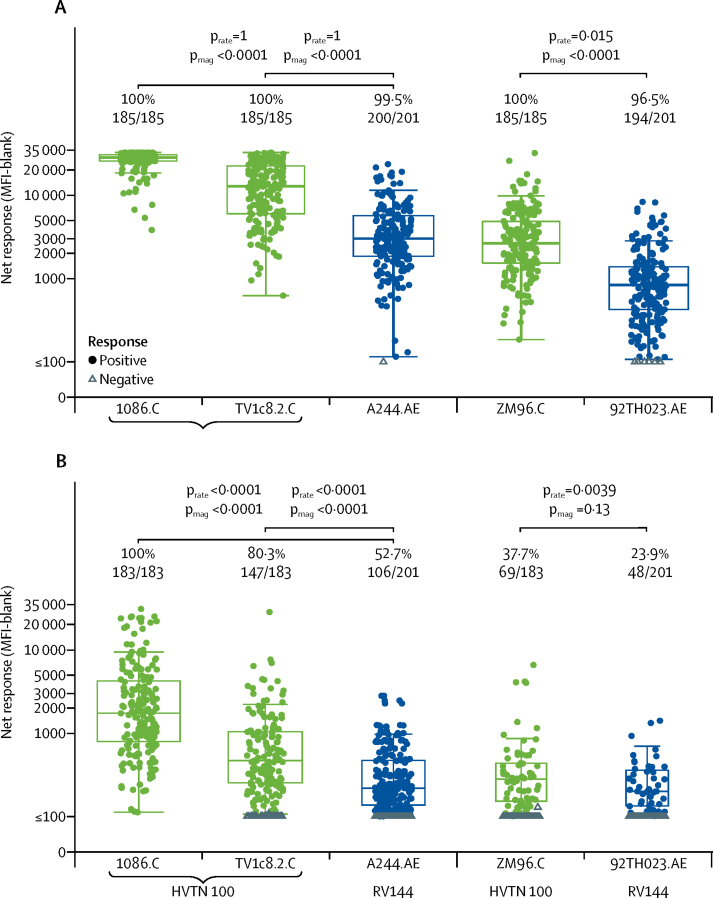
Binding antibody responses to env gp120 vaccine-insert antigens among vaccine recipients at month 6·5 Boxplots show (A) IgG and (B) IgG3 responses and are based on positive responders only (shown as coloured circles); negative responders are shown as grey triangles and positive response rates are indicated above the boxes. p values compare response rates (p_rate_) and magnitudes (p_mag_) among positive responders between HVTN 100 and RV144 vaccine recipients. env=envelope glycoprotein. MFI=mean fluorescence intensity.

185 (100%) HVTN 100 vaccine recipients in the per-protocol cohort developed IgG binding antibodies to all three subtype C gp120 vaccine-matched env insert antigens (1086.C, ZM96.C, and TV1c8.2.C; figure 2A, table 2); no positive responses were seen among placebo recipients (data not shown). The response rate for each antigen was 100% (95% CI 98–100; figure 1). Among 201 vaccine recipients in RV144, IgG antibody response rates to vaccine-matched env gp120 antigens were also high, with 200 (99·5%, 95% CI 97·2–99·9) participants having a response to A244.AE and 194 (96·5%, 95% CI 93·0–98·3) having a response to 92TH023.AE (figure 2A, table 2). Among positive responders, the magnitude of IgG binding antibody responses in HVTN 100 to both 1086.C and TV1c8.2.C was significantly higher than those seen in RV144 positive responders to A244.AE (both p<0·0001; figure 2A). Similarly, the magnitude of responses to ZM96.C in HVTN 100 was significantly higher than to the corresponding vaccine-matched antigen, 92TH023.AE, in RV144 (p<0·0001; figure 2A). The GMTs of IgG binding antibodies to vaccine-matched insert antigens were 3·6-times (95% CI 3·0–4·4) to 8·8-times (95% CI 7·6–10·2) higher in HVTN 100 than in RV144 vaccine recipients with corresponding vaccine-matched antigens (figure 1).

**Table 2:** IgG and IgG3 binding antibody, intracellular cytokine staining, and neutralising antibody responses among vaccine recipients at month 6·5

		**HVTN 100 response rate (%)**		**RV144 response rate (%)**		**p value**
		n/N	% (95% CI)	n/N	% (95% CI)	
**Binding antibody multiplex assay**						
IgG						
	Any gp120*	185/185	100% (98·0–100)	199/201	99·0% (96·4–99·7)	0·50
	1086.C gp120 (HVTN 100) *vs* A244.AE gp120 (RV144)†	185/185	100% (98·0–100)	200/201	99·5% (97·2–99·9)	1·00
	TV1c8.2.C gp120 (HVTN 100) *vs* A244.AE gp120 (RV144)†	185/185	100% (98·0–100)	200/201	99·5% (97·2–99·9)	1·00
	ZM96.C gp120 (HVTN 100) *vs* 92TH023.AE gp120 (RV144)†	185/185	100% (98·0–100)	194/201	96·5% (93·0–98·3)	0·015
	Con 6 gp120/B‡	181/181	100% (97·9–100)	199/201	99·0% (96·4–99·7)	0·50
	Any V1V2§	174/183	95·1% (90·9–97·4)	200/201	99·5% (97·2–99·9)	0·0080
	Any subtype C V1V2¶	153/183	83·6% (77·6–88·3)	199/201	99·0% (96·4–99·7)	<0·0001
	1086_V1V2_Tags.C†	129/183	70·5% (63·5–76·6)	199/201	99·0% (96·4–99·7)	<0·0001
	CaseA2_gp70_V1V2.B†	91/183	49·7% (42·6–56·9)	160/201	79·6% (73·5–84·6)	<0·0001
	TV1.21.C V1V2†	113/183	61·7% (54·5–68·5)	167/201	83·1% (77·3–87·6)	<0·0001
	TV1c8.2.C V1V2	111/178	62·4% (55·1–69·1)	..	..	..
	gp70-ConC V1V2‡	89/170	52·4% (44·9–59·7)	..	..	..
IgG3						
	Any gp120‖	183/183	100% (97·9–100)	157/201	78·1% (71·9–83·3)	<0·0001
	Con 6 gp120/B	116/183	63·4% (56·2–70·0)	53/201	26·4% (20·8–32·9)	<0·0001
	Any V1V2**	64/183	35·0% (28·4–42·1)	160/201	79·6% (73·5–84·6)	<0·0001
	Any subtype C V1V2††	51/183	27·9% (21·9–34·8)	108/201	53·7% (46·8–60·5)	<0·0001
	gp70-ConC V1V2	10/181	5·5% (3·0–9·9)	..	..	..
**Intracellular cytokine staining**						
IL-2 or IFN-γ or CD40L CD4+ T cells						
	Any gp120‡‡	120/181	66·3% (59·1–72·8)	..	..	..
	env.ZM96.C (HVTN 100) *vs* env.92TH023.AE (RV144)†	102/181	56·4% (49·1–63·4)	79/191	41·4% (34·6–48·4)	0·005
IL-2 or IFN-γ or IL-2 and IFN-γ CD4+ T cells						
	Any gp120‡‡	97/179	54·2% (46·9–61·3)	..	..	..
	env.ZM96.C (HVTN 100) *vs* env.92TH023.AE (RV144)	87/179	48·6% (41·4–55·9)	71/195	36·4% (30·0–43·4)	0·021
**TZM-Bl neutralising antibodies**						
Any vaccine-matched isolate§§		182/185	98·4% (95·3–99·4)	..	..	..
MW965.26.C		183/185	98·9% (96·1–99·7)	..	..	..
Any subtype C Tier 1A¶¶		183/185	98·9% (96·1–99·7)	..	..	..
Any subtype C Tier 1B‖‖		83/185	44·9% (37·9–52·1)	..	..	..

env=envelope protein. IFN=interferon. IL=interleukin. V1V2=variable loops 1 and 2.

* 1086C_D7gp120.avi/293F, TV1c8_D11gp120.avi/293F, 96ZM651.D11gp120.avi (HVTN100) or 92TH023 gp120 gDneg 293F mon (RV144).

† Prespecified immunological criterion.

‡ IgG responses to Con 6 gp120/B (1:100) and gp70-ConC V1V2 (1:50) were tested at lower dilutions than other gp120 antigens (1:200) and V1V2 antigens (1:100).

§ C.1086_V1_V2 Tags, gp70-001428.2.42 V1V2, gp70-7060101641 V1V2, gp70-96ZM651.02 V1v2, gp70-BF1266_431a_V1V2, gp70-CAP210.2.00.E8 V1V2, gp70-TV1.21 V1V2, gp70_B.CaseA_V1_V2, gp70_B.CaseA2 V1/V2/169K, gp70-62357.14 V1V2, gp70-191084_B7 V1V2, gp70-700010058 V1V2, gp70-C2101.c01_V1V2, gp70-BJOX002000.03.2, gp70-CM244.ec1 V1V2, gp70-RHPA4259.7 V1V2, gp70-TT31P.2F10.2792 V1V2 (same antigens for both trials).

¶ C.1086_V1_V2 Tags, gp70-001428.2.42 V1V2, gp70-7060101641 V1V2, gp70-96ZM651.02 V1v2, gp70-BF1266_431a_V1V2, gp70-CAP210.2.00.E8 V1V2, gp70-TV1.21 V1V2 (same antigens for both trials).

‖ 1086C_D7gp120.avi/293F, TV1c8_D11gp120.avi/293F, A244 D11gp120_avi, Con 6 gp120/B, 96ZM651.D11gp120.avi (HVTN100) or 92TH023 gp120 gDneg 293F mon (RV144).

** AE.A244 V1V2 Tags/293F, C.1086_V1_V2 Tags, gp70-96ZM651.02 V1v2, gp70-TV1.21 V1V2, gp70_B.CaseA_V1_V2, gp70_B.CaseA2 V1/V2/169K (same antigens for both trials).

†† C.1086_V1_V2 Tags, gp70-96ZM651.02 V1v2, gp70-TV1.21 V1V2.

‡‡ env.1086.C, env.TV1.C, Env.ZM96.C (HVTN 100); only Env.92TH23.AE measured in RV144 so not included.

§§ 96ZM651.2, Ce1086_B2, TV1c8.2.

¶¶ CH0505.w4.3, MW965.26, SO032_A2.8-1.

‖‖ 1107356.07, 6644.v2.c33, CA146 H3.3.

**Figure 3:**
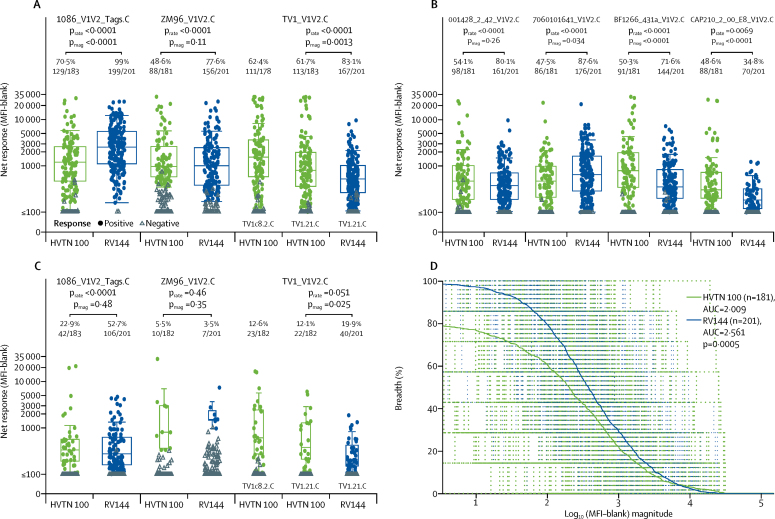
Binding antibody response rates to V1V2 antigens among vaccine recipients at month 6·5 Boxplots show (A and B) IgG and (C) IgG3 responses to vaccine-matched antigens and are based on positive responders only (shown as coloured circles); negative responders are shown as grey triangles and positive response rates are indicated above the boxes. p values compare response rates (p_rate_) and magnitudes (p_mag_) among positive responders between HVTN 100 and RV144 vaccine recipients. (D) Plot shows the magnitude–breadth of IgG binding antibody responses to subtype C env V1V2 antigens among vaccine recipients in the per-protocol cohorts of HVTN 100 and RV144 2 weeks after the month 6 vaccination. Solid curves are average breadth across individuals for HVTN 100 and RV144 vaccine recipients. Breadth is defined as the proportion of antigens in the panel with log_10_ (MFI – blank) greater than the threshold on the *x* axis. AUC=area under the curve. env=envelope glycoprotein. MFI=mean fluorescence intensity. V1V2=variable loops 1 and 2.

Among 183 HVTN 100 vaccine recipients, the IgG binding antibody response rate to the three prespecified immunological criteria V1V2 antigens was highest to the 1086_V1V2_Tags.C strain in the gp120 boost vaccine (70·5%, 95% CI 63·5–76·6; n=129 responders), followed by TV1.21.C (61·7%, 95% CI 54·5–68·5; n=113 responders) and CaseA2_gp70_V1V2.B (49·7%, 95% CI 42·6–56·9; n=91 responders; figure 1, table 2). A V1V2 response to any of the seven subtype C V1V2 antigens considered was noted in 153 (83·6%) of 183 vaccine recipients in HVTN 100 (95% CI 77·6–88·3; table 2). In RV144 vaccine recipients, IgG binding antibody response rates to all V1V2 antigens except CAP210_2_00_E8_V1V2.C were significantly higher than those in HVTN 100 vaccine recipients (figure 3A, 3B). The breadth of responses to a panel of subtype C V1V2 antigens was significantly higher in RV144 than HVTN 100 vaccine recipients (p=0·0005; figure 3D). We did not see positive V1V2 responses to subtype C antigens among placebo recipients in either trial (data not shown).

Among 183 per-protocol vaccine recipients in HVTN 100, IgG3 binding antibody responses to gp120 boost env antigens were significantly higher in response rate and magnitude among positive responders compared with responses to the corresponding antigen in 201 patients in RV144 (all p<0·0001; figure 2B). The response rate to 1086.C was 100% (95% CI 97·9–100; n=183 responders) followed by 80·3% to TV1c8.2.C (95% CI 74·0–85·4; n=147 responders), compared with 52·7% to A244.AE in RV144 (95% CI 45·8–59·5; n=106 responders). The response rate to the ALVAC-HIV insert env ZM96.C in HVTN 100 was 37·7% (95% CI 31·0–44·9; n=69 responders), which is significantly higher than the response rate of 23·9% (95% CI 18·5–30·2; n=48 responders) for the corresponding ALVAC-insert env 92TH023.AE in RV144 (p=0·0039; figure 2B). For the V1V2 region, the IgG3 response rate to the 1086_V1V2_Tags.C antigen was significantly higher in vaccine recipients in RV144 (52·7%, 95% CI 45·8–59·5; n=106 responders) than in HVTN 100 (22·9%, 95% CI 17·5–29·6; n=42 responders; p<0·0001; figure 3C). However, no significant differences were seen in magnitude among positive responders to the 1086_V1V2_Tags.C antigen or in the response rates or magnitude among positive responders to the ZM96_V1V2.C antigen (figure 3C). For the TV1.21.C V1V2 antigen, the magnitude among positive responders was higher among vaccine recipients in HVTN 100 than in RV144 (p=0·025), although there was a trend towards a higher response rate for RV144 (p=0·051; figure 3C).

Although responses to serum binding antibody multiplex assays (measured in HVTN 100) are of slightly higher magnitude (at most, 0·10 log_10_ net mean fluorescence intensity) than are plasma responses (measured in RV144) for some gp120 antigens, differences for V1V2 antigens were not recorded (appendix p 8). The gp120 and V1V2 results from the sensitivity analysis were nearly identical to the main findings.

**Figure 4:**
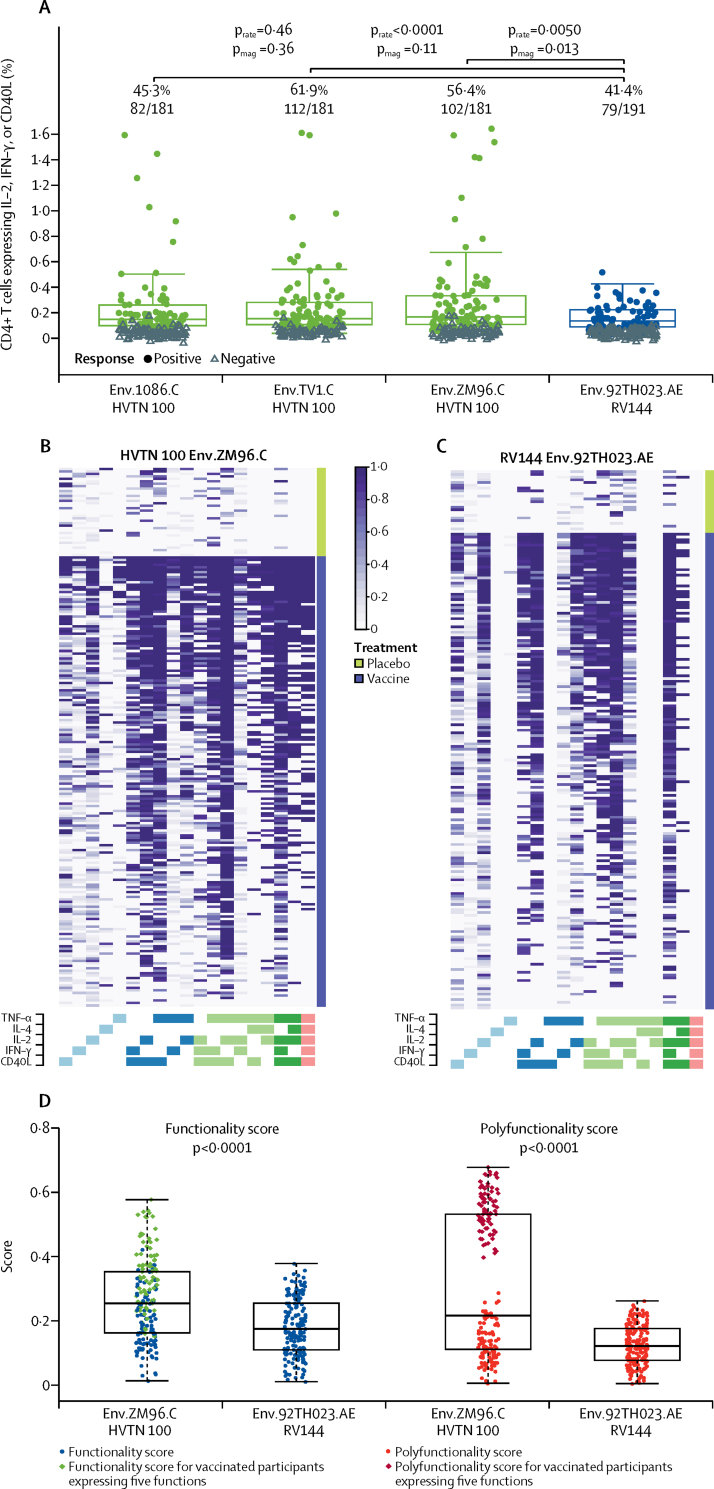
CD4+ T-cell responses to vaccine-matched env antigens among vaccine recipients at month 6·5 (A) Boxplots show expression of interleukin-2, interferon-γ, or CD40L by CD4+ T cells and are based on positive responders only (shown as coloured circles); negative responders are shown as grey triangles and positive response rates are indicated above the boxes. p values compare response rates (p_rate_) and magnitudes (p_mag_) among positive responders between HVTN 100 and RV144 vaccine recipients. One participant had a positive response to env.TV1.C gp120 greater than 2%. (B and C) Heatmaps of COMPASS posterior probabilities for CD4+ T-cell subsets to env antigens. Columns correspond to the different cell subsets, identified by the blue, green, and pink grid that indicates 19 of 32 possible subsets with env-specific responses detectable in more than five cells and in more than two participants in HVTN 100. Purple and white colour-coding indicates the expression of cytokines (white indicates the subset is not expressed, purple shading indicates it is expressed), ordered by degree of functionality from one function on the left (light blue) to five functions on the right (pink). Rows correspond to participants, ordered by treatment group (placebo or vaccine), and by functionality score within each group. Each cell of the heatmap shows the probability that the corresponding cell-subset (column) shows an antigen-specific response in the corresponding participant (row), where the probability is colour-coded from white (zero) to purple (one). (D) Functionality and polyfunctionality scores of CD4+ T-cell subsets recognising env antigens. COMPASS=combinatorial polyfunctionality analysis of antigen-specific T-cell subsets. env=envelope glycoprotein. IFN=interferon. IL=interleukin.

Among 181 vaccine recipients in HVTN 100, 102 had a response for CD4+ T cells expressing interleukin-2, interferon-γ, or CD40L specific for the vaccine-matched env insert ZM96.C (56·4%, 95% CI 49·1–63·4) compared with 79 of 191 vaccine recipients in RV144 having a response to the corresponding env insert 92TH023.AE (41·4%, 95% CI 34·6–48·4; difference in response 15·0%, 95% CI 4·8–24·9; p=0·0050; table 2, figure 4A). The magnitude of the positive responders of CD4+ T cells expressing interleukin-2, interferon-γ, or CD40L was significantly higher among vaccine recipients in HVTN 100 than in RV144 (p=0·013; figure 4A), with functionality and polyfunctionality scores also higher (both p<0·0001; figure 4D). We did not see any positive responses among placebo recipients (data not shown). Moreover, vaccine recipients in HVTN 100 had a different profile of polyfunctional CD4+ T cells (figure 4B, 4C, 4D) with significantly higher responses for the tumour necrosis factor (TNF)-α+ interleukin-2+ CD40L+ T-cell subset, the TNF-α+ interleukin-2+ interleukin-4+ CD40L+ T-cell subset, and the TNF-α+ interleukin-2+ interleukin-4+ interferon-γ+ CD40L+ T-cell subset (all p<0·0001, appendix p 8).

All prespecified immunological criteria were met (figure 1). No antibody-mediated neutralisation was detected against the HIV-1 subtype C Tier 2 vaccine strains or the related TV1.21.C virus. Antibodies induced in HVTN 100 vaccine recipients neutralised isolates showing a highly sensitive Tier 1A and Tier 1B neutralisation phenotype. Responses were strongest against the heterologous Tier 1A strain MW965.26.C (98·9%, 95% CI 96·1–99·7; n=183 responders) and the Tier 1A vaccine strain TV1c8.2.C (98·4%, 95% CI 95·3–99·4; n=182 responders; table 2). Less frequent responses of low-to-moderate potency were seen against other Tier 1A and Tier 1B subtype C isolates (appendix p 4). Neutralising antibody assays were not done contemporaneously on the RV144 plasma samples.

HVTN 100 and RV144 vaccine recipients both displayed distinct immunological profiles, as shown by the individual response clustering by trial (appendix p 5). The differences are driven by higher IgG and IgG3 env gp120 binding and intracellular cytokine staining responses seen in HVTN 100 and higher IgG V1V2 responses seen in RV144, as indicated by the distribution of HVTN 100 and RV144 datapoints present near each respective response arrow in the biplot (appendix p 5). HVTN 100 vaccine recipients also clustered distinctly from placebo recipients, indicating good specificity of the immunogenicity assays (appendix p 6). We saw high correlations in responses to different antigens within each assay (eg, Spearman correlations between IgG responses to the env gp120 vaccine insert antigens ranged from 0·77 to 0·91), with generally weaker correlations seen across assays (appendix pp 5, 6). Few HVTN 100 vaccine recipients had negative responses across multiple assays: only one (1%) of 175 participants receiving the vaccine was negative for the combination of humoral and cellular responses tested here.

## Discussion

Our study findings show that an HIV-1 subtype C vaccine regimen using the canarypox prime and gp120 env protein boost strategy of the RV144 vaccine regimen induced IgG V1V2 responses and significantly higher CD4+ T-cell responses and gp120 binding antibody responses compared with the RV144 regimen, enabling assessment of these variables as potential correlates of protection in a follow-up efficacy trial. A multivariate analysis of binding, neutralisation, and cellular responses showed that the HVTN 100 regimen induced a different immunological profile compared with RV144, indicating the potential effect of subtype-specific vector-based gene inserts and proteins, adjuvants, or both on immune responses after HIV vaccination. Further studies are needed to address whether the breadth of vaccine-induced anti-gp120 binding antibodies is related to recognition of conserved epitopes among multiple isolates or multiple strain-specific epitopes, or both. We did not identify any safety concerns with the HVTN 100 regimen and immune responses exceeded the prespecified prevalence and titre for both antibody and CD4+ T-cell responses established to move this regimen forward into efficacy trials. On the basis of these results, a phase 2b/3 trial of this regimen (HVTN 702) began enrolling in South Africa in October, 2016.

Findings of correlate-of-risk studies done after the RV144 results were released in 2009 indicated that a reduced risk of HIV infection was associated with higher levels of binding antibodies to HIV env (total IgG and IgG3) and antibodies to scaffolded antigens, including the V1V2 loop.^10^, ^13^, ^19^ High CD4+ T-cell functionality and polyfunctionality scores to HIV-1 env antigens were also associated with decreased risk of HIV infection.^12^ Our study findings show that some immune responses associated with decreased risk of HIV infection in RV144 were higher in HVTN 100 than in RV144. Moreover, IgG3 responses to these env gp120 antigens were of a greater frequency in HVTN 100 than in RV144. Because the gp120 IgG binding responses in RV144 to vaccine-matched env glycoproteins (ie, A244 gp120 and 92TH023 gp120) were correlates of decreased infection risk,^10^ and the level of V1V2 antibody prevalence exceeded the threshold associated with 50% vaccine efficacy, these results offer cautious optimism for future efficacy of this regimen. However, the higher incidence of HIV in South Africa compared with Thailand might raise the threshold for protection. Moreover, an ALVAC and MF59-adjuvanted gp120 simian immunodeficiency virus (SIV) vaccine regimen had no efficacy in macaques by contrast with an ALVAC and alum-adjuvanted gp120 SIV vaccine regimen.^28^ Whether the subtype C-adapted vaccine regimen tested here is efficacious remains to be assessed in the ongoing HVTN 702 phase 2b/3 trial.

What is the role of and interaction between T-cell and antibody responses in HIV vaccine development? Detailed analyses of CD4+ T-cell responses in the RV144 trial showed that vaccine recipients with a high magnitude of env-specific CD4+ T cells measured by intracellular cytokine analyses had a lower risk of HIV infection. In particular, vaccine recipients whose CD4+ T cells exposed to HIV-1 env antigen produced multiple cytokines (TNF-α, interferon-γ, interleukin-4, interleukin-2, and CD40L [CD154]) had a lower risk of HIV infection.^12^ env-specific CD4+ T-cell responses were of higher prevalence and magnitude in HVTN 100 than in RV144, with the highest responses noted with the TV1c8.2.C antigen, suggesting that recombinant protein immunogens are important inducers of CD4+ T-cell responses. 11 vaccine recipients in HVTN 100 generated ex-vivo env-specific CD4+ T-cell frequencies greater than 0·5% when assessed by flow cytometric expression of interleukin-2, interferon-γ, or CD40L, with some responses as high as 1·5%. No vaccine recipient in RV144 had intracellular cytokine staining responses greater than 0·4%. Moreover, T-cell functionality and polyfunctionality scores were significantly higher in HVTN 100. Single-cell analysis of the cytokine secretion pattern of CD4+ T cells to HIV-1 env showed that the expected probability of response to the vaccine-matched env insert for cells expressing five cytokines (TNF-α, interferon-γ, interleukin-4, interleukin-2, and CD40L) was 43·6% for vaccine recipients in HVTN 100 compared with a probability of 0% among the RV144 vaccine recipients assessed in this study. Whether these differences can be attributed to the selected vaccine strains that are more highly immunogenic than those in RV144, or to the MF59 adjuvant rather than the alum adjuvant, is unclear. Because MF59 induces interleukin-4 responses better than alum,^29^ the MF59 adjuvant used in our regimen might have contributed to the greater functionality seen in HVTN 100. MF59 has been approved in Europe to enhance the immunogenicity of seasonal influenza vaccine among elderly people.^30^ Furthermore, influenza vaccine formulated with MF59 significantly enhances immune responses and efficacy in young children.^31^

The HVTN 100 regimen induced a lower response than the RV144 vaccine regimen in the generation of antibodies to the env V1V2 region. Although most HVTN 100 recipients developed antibodies to V1V2 subtype C proteins after vaccination, significantly higher GMTs were seen to several V1V2 antigens with use of the RV144 regimen. This difference is unlikely to be attributable to characteristics of the adult Thai and South African populations. When the RV144 regimen was given in South Africa (HVTN 097), IgG binding antibody responses to V1V2 were similar to those noted in RV144;^32^ thus differences in V1V2 antibody responses between HVTN 100 and HVTN 097 are probably attributable to antigenic subtype differences between the proteins. Several monoclonal antibodies to the V1V2 region that were isolated from recipients receiving the RV144 vaccine bound well to strain 1086.C but not to TV1.C in the HVTN 100 vaccine regimen, suggesting that antigenic differences in env gp120 might contribute to the differences in V1V2 immunogenicity. Despite these discrepancies, the cumulative HVTN 100 V1V2 response to any clade C antigen considered was 83·6%, well above the established 63% threshold for predicting a vaccine efficacy of 50% at 2 years if V1V2 response status was the sole correlate of protection.^33^

While we succeeded in satisfying the primary study objective, to qualify this vaccine regimen for efficacy evaluation based on a predetermined set of criteria, this report is necessarily restricted to the primary immune response data. Additional goals of the study—including the final unblinded safety evaluation, immunogenicity analyses of cross-clade breadth, functional immune responses, effects of subsequent boosting vaccinations, and durability—will be reported once the study has been completed. Further, the generalisability of these results to other HIV vaccine platforms may be limited.

Our findings show that the subtype C vaccine regimen of a recombinant canarypox vector containing a subtype C env antigen in combination with an MF59-adjuvanted subtype C bivalent gp120 protein vaccine induced strong humoral and cellular immune responses and an immune profile distinct from that observed in RV144. Further studies are needed in vaccine recipients to assess the mechanisms underlying immune heterogeneity in response to vaccination, particularly those with low or no response. The ongoing HVTN 702 efficacy trial will assess whether immune responses extend to vaccine efficacy in subtype C-endemic regions.
