# Supplementary material for: Subtype C ALVAC-HIV and bivalent subtype C gp120/MF59 HIV-1 vaccine in low-risk, HIV-uninfected, South African adults: a phase 1/2 trial
Source: Lancet HIV. 2018 Jun 18;5(7):e366–78. doi: 10.1016/S2352-3018(18)30071-7 (PMC6028742; doi:10.1016/S2352-3018(18)30071-7)
Supplement: Supplementary appendix [file mmc1.pdf]

THE LANCET
HIV

Supplementary appendix

This appendix formed part of the original submission and has been peer reviewed. We post it as supplied by the authors.

Supplement to: Bekker L–G, Moodie Z, Grunenberg N, et al, on behalf of the HVTN 100 Protocol Team. Subtype C ALVAC-HIV and bivalent subtype C gp120/MF59 HIV-1 vaccine in low-risk, HIV-uninfected, South African adults: a phase 1/2 trial. *Lancet HIV* 2018; published online June 10. http://dx.doi.org/10.1016/S2352-3018(18)30071-7.

Supplementary material for "A phase 1/2 HIV-1 trial of a Subtype C ALVAC-HIV and Bivalent Subtype C gp120/MF59 vaccine regimen in low risk HIV uninfected South African adults" by Bekker et al.

Table of Contents

Details of the pre-specified immunological criteria ... 2

Expanded methods for immunological assays ... 2

HVTN Protocol Team listing ... 3

Supplementary Figure 1: Neutralising antibody responses to clade C isolates among vaccine recipients in the per-protocol cohort of HVTN 100 two weeks after the Month 6 vaccination. ... 4

Supplementary Figure 2: Multi-assay principal components analysis (PCA) biplot (Panel A) and Spearman correlation heatmap (Panel B) for vaccine recipients in the per-protocol cohorts of HVTN 100 and RV144 two weeks after the Month 6 vaccination. ... 5

Supplementary Figure 3: Multi-assay principal components analysis (PCA) biplot (Panel A) and Spearman correlation heatmap (Panel B) for vaccine and placebo recipients in the per-protocol cohort of HVTN 100 two weeks after the Month 6 vaccination. ... 6

Supplementary Table 1: Details of the BAMA, ICS, and nAb antigens including HIV-1 viral strain information ... 7

Supplementary Table 2: Expected probabilities of response for common HVTN 100 CD4+ T cell subsets to vaccine-matched Env antigens among vaccine recipients in the per-protocol cohorts of HVTN 100 and RV144 two weeks after the Month 6 vaccination, estimated as the average of the individual-specific estimated probabilities that the given T cell subset is positive. ... 8

Supplementary Table 3: Mean differences, 95% CIs, and paired t-test p-values comparing BAMA responses in serum vs plasma in HVTN097 samples. ... 8

Supplementary Text

Details of the pre-specified immunological criteria

The primary immunogenicity endpoints of the pre-specified immunological criteria (Figure 1) were (i and ii) response rate and magnitude of vaccine-induced IgG antibody binding to proteins covering the three gp120 Env strains contained in the vaccine regimen (1086.C, TV1c8.2.C, and ZM96.C); (iii) vaccine-induced $CD4^+$ T-cell responses to the HIV Env included in the vector (Env.ZM96.C); and (iv) vaccine-induced IgG antibody binding to V1V2 Env proteins (1086_V1V2_Tags.C, TV1.21.C, CaseA2_gp70_V1V2.B). The subtype of the antigen or pseudovirus is indicated by the last letter of the name (e.g., 1086.C).

The original clone for the TV1.C vaccine was TV1c8.2.C; however, the V1V2 antigen designated for the pre-specified immunological criteria was TV1.21.C. For HVTN 100, binding antibody responses to both antigens were measured; however, only responses to the TV1.21.C antigen were measured for RV144 samples.

Expanded methods for immunological assays

Intracellular Cytokine Staining (ICS) to measure Env-specific CD4+ T cell response

Two different marker subsets were analysed for each of the antigens in Table S1: ‘IL2 or IFN-γ or CD40L’ and ‘IL2 and/or IFN-γ’. For each of these subsets, the magnitude of response, or “net response”, is the difference between the stimulated and the average of the two unstimulated wells of the percent of CD4+ T cells that express at least one of the markers in the subset. This percent was calculated as the sum of the cell counts across all Boolean combinations of the markers divided by the total number of CD4+ T cells.

For the 3 marker subset ‘IL2 or IFN-γ or CD40L’, the positive response definition described in the next paragraph was applied to the marginal data for each of the 3 markers, and the overall response is positive if any of the 3 marginal responses were positive. For the 2 marker subset ‘IL2 and/or IFN-γ, the positive response definition was applied to the aggregate data for the 2 markers. The filtering described above was applied to the marginal data for the 3 marker subset and applied to the aggregate data for the 2 marker subset. The response based on 3 marginal markers was filtered if the sample was filtered for any of the 3 marginal markers.

Positivity for a peptide pool within a T-cell subset was determined by a one-sided Fisher's exact test applied to the peptide pool-specific response versus the negative control response with no multiplicity adjustment since only a single peptide pool was considered for each trial. Peptide pools with p-values less than $\alpha = 0{\cdot}00001$ were considered positive.[1]

Binding antibody multiplex assay (BAMA) to measure binding antibody (bAb) response

The gp120 and V1V2 antigens assessed with BAMA are included in Supplementary Table 1. The readout was background-subtracted mean fluorescence intensity (MFI), where background accounts for both an antigen-specific plate level control (i.e., a blank well containing antigen-coated beads run on each plate), and a specimen-specific control (i.e., a serum well containing blank beads). The positive controls were purified polyclonal IgG from HIV-positive subjects (HIVIG) using a 10-point standard curve (4PL fit) and CH58 mAb titration. The negative controls were NHS (HIV-1 sero-negative human sera) and blank beads. The sample was repeated if the blank bead negative control exceeded 5000 MFI. If the repeat value exceeded 5000 MFI, the sample was excluded from analysis due to high background. The MFI minus Blank bead responses (“net MFI”) at the specified dilutions are used to summarise the magnitude. Net MFI less than 1 was set to 1.

Samples were declared positive if the following held: (1) net MFI ≥ antigen-specific positive response threshold (defined separately for each trial as the maximum of 100 and the

---

[1] Horton, H. *et al.* Optimization and validation of an 8-color intracellular cytokine staining (ICS) assay to quantify antigen-specific T cells induced by vaccination. *J Immunol Methods* **323**, 39-54, doi:10.1016/j.jim.2007.03.002 (2007).

95th percentile of pre-vaccination net MFI values), (2) net MFI > 3 times baseline net MFI, and (3) MFI > 3 times baseline MFI.

TZM-bl assay to measure neutralising antibody (nAb) responses

The TZM-bl assay measured neutralising antibody titres against the HIV-1 viruses listed in Supplementary Table 1.

HVTN 100 Protocol Team

Linda-Gail Bekker, Dorothie Van der Vendt, Mluleki Nompondwana, Desmond Tutu HIV Centre, Institute of Infectious Disease and Molecular Medicine, University of Cape Town, Cape Town, South Africa
Fatima Laher, Perinatal HIV Research Unit, Chris Hani Baragwanath Hospital, Soweto, South Africa
Nicole Grunenberg, Zoe Moodie, Simba Takuva, Eva Chung, On Ho, John Hural, Erica Andersen-Nissen, Elizabeth Briesemeister, Jill Zeller, Carissa Karg, Huguette Redinger, Jennifer Schille, Gina Escamilla, Shannon Grant, Carter Bentley, Genevieve Meyer, Erik Schwab, April Randhawa, Adi Ferrara. Vaccine and Infectious Disease Division, Fred Hutchinson Cancer Research Center, Seattle, Washington, USA
Mary Allen, Chuka Anude, Scharla Estep, Katherine Shin, Michael Pensiero, Division of AIDS, National Institute of Allergy and Infectious Diseases, National Institutes of Health, Bethesda, Maryland, USA
Georgia Tomaras, Duke Human Vaccine Institute, Durham, North Carolina, USA
Carlos DiazGranados, Sanjay Phogat, Sanofi Pasteur, Swiftwater, Pennsylvania, USA
Susan Barnett, Niranjan Kanesa-thasan, Novartis Vaccines and Diagnostics, Cambridge, Massachusetts, USA
Marguerite Koutsoukos, Olivier van der Meeren, Francois Roman, GlaxoSmithKline, Rixensart, Belgium
Jerry Phore, HVTN Community Advisory Board, Klerksdorp, South Africa
Goduka Mfana, HVTN Community Advisory Board, Soweto, South Africa
Mzwandile Phanziso, HVTN Community Advisory Board, Cape Town, South Africa

Supplementary Figure 1: Neutralising antibody responses to clade C isolates among vaccine recipients in the per-protocol cohort of HVTN 100 two weeks after the Month 6 vaccination. Boxplots are based on positive responders only with negative responders shown in grey triangles with positive response rates above the boxes.

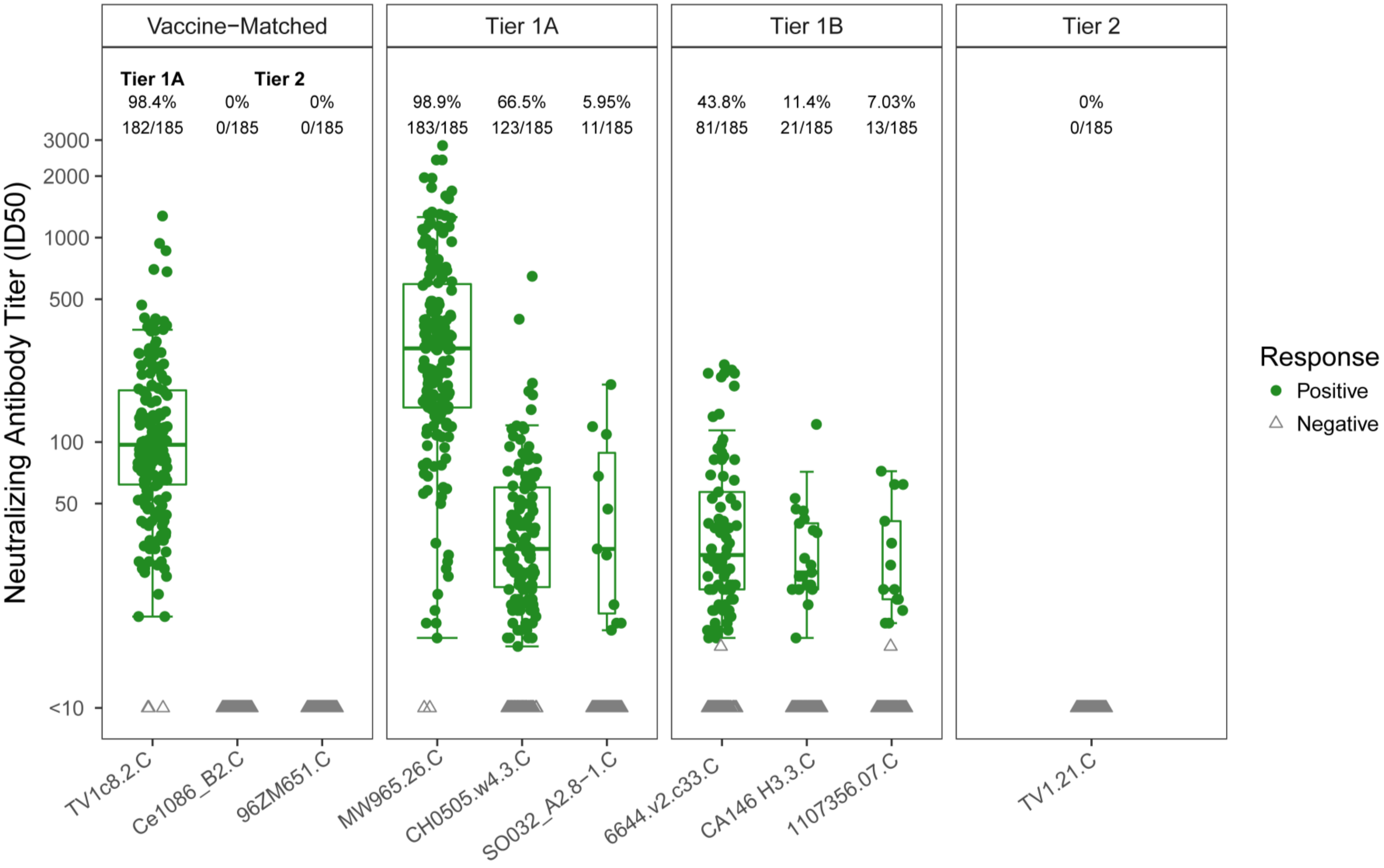

Supplementary Figure 2: Multi-assay principal components analysis (PCA) biplot (Panel A) and Spearman correlation heatmap (Panel B) for vaccine recipients in the per-protocol cohorts of HVTN 100 and RV144 two weeks after the Month 6 vaccination.

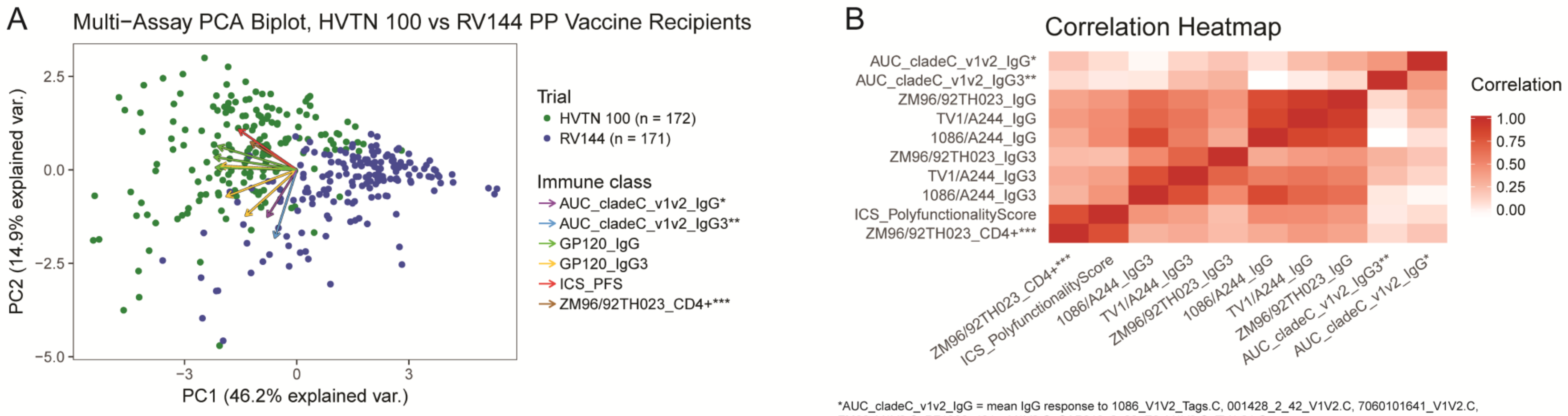

*AUC_cladeC_v1v2_IgG = mean IgG response to 1086_V1V2_Tags.C, 001428_2_42_V1V2.C, 7060101641_V1V2.C, ZM96_V1V2.C, BF1266_431a_V1V2.C, CAP210_2_00_E8_V1V2.C, TV1.21.C
**AUC_cladeC_v1v2_IgG3 = mean IgG response to 1086_V1V2_Tags.C, TV1.21.C, ZM96_V1V2.C
***ZM96/92TH023_CD4+ = ICS IL2/IFNg response to ZM96 (HVTN 100) or 92TH023 (RV144)

Supplementary Figure 3: Multi-assay principal components analysis (PCA) biplot (Panel A) and Spearman correlation heatmap (Panel B) for vaccine and placebo recipients in the per-protocol cohort of HVTN 100 two weeks after the Month 6 vaccination.

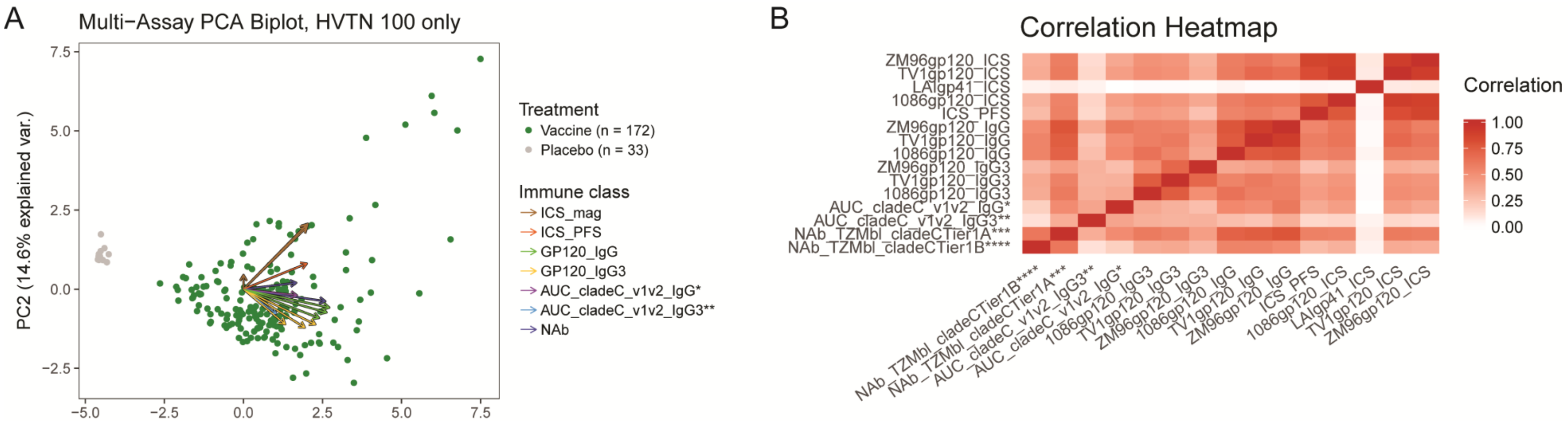

*AUC_cladeC_v1v2_IgG = mean IgG response to 1086_V1V2_Tags.C, 001428_2_42_V1V2.C, 7060101641_V1V2.C, ZM96_V1V2.C, BF1266_431a_V1V2.C, CAP210_2_00_E8_V1V2.C, TV1.21.C

**AUC_cladeC_v1v2_IgG3 = mean IgG response to 1086_V1V2_Tags.C, TV1.21.C, ZM96_V1V2.C

***NAb_TZMbl_cladeCTier1A = CH0505.w4.3.C, MW965.26.C, SO032_A2.8-1.C

****NAb_TZMbl_cladeCTier1B = 1107356.07.C, 6644.v2.c33.C, CA146 H3.3.C

Supplementary Table 1: Details of the BAMA, ICS, and nAb antigens including HIV-1 viral strain information

Assay	Antigen class	Full antigen name	Antigen label used in plot	Viral strain information: Subtype.Country.Year.Stage*
BAMA bAb	gp120	1086C_D7gp120.avi/293F	1086.C	C.MW.04.1-2
		TV1c8_D11gp120.avi/293F	TV1c8.2.C	C.ZA.98.6
		96ZM651.D11gp120.avi	ZM96.C	C.ZM.96.6
		A244 D11gp120_avi	A244.AE	CRF01_AE.TH.90.6
		92TH023 gp120 gDneg 293F mon	92TH023.AE	CRF01_AE.TH.92.6
		Con 6 gp120/B	Con 6 gp120/B	-
	V1V2	C.1086_V1_V2 Tags	1086_V1V2_Tags.C	C.MW.04.1-2
		gp70_B.CaseA_V1_V2	CaseA2_gp70_V1V2.B	B.US.88.6
		gp70-TV1.21 V1V2	TV1.21.C	C.ZA.98.6
		gp70-TV1.GSKvacV1V2/293F	TV1c8.2.C	C.ZA.98.6
		gp70-96ZM651.02 V1v2	ZM96_V1V2.C	C.ZM.96.6
		gp70-001428.2.42	001428_2_42_V1V2.C	C.IN.00.4
		gp70-7060101641 V1V2	7060101641_V1V2.C	C.ZA.07.3
		gp70-BF1266_431a_V1V2	BF1266_431a_V1V2.C	C.MW.02.1-2
		gp70-CAP210.2.00.E8 V1V2	CAP210_2_00_E8_V1V2.C	C.ZA.05.4
		gp70-B.CaseA2 V1/V2/169K	B.CaseA2_V1/V2/169K.B	B.US.88.6
		gp70-62357.14 V1V2	62357_14.V1V2.B	B.US.96.2
		gp70-191084_B7 V1V2	191084_B7.V1V2.A	A1.UG.07.4
		gp70-700010058 V1V2	700010058.V1V2.B	B.US.06.3
		gp70-C2101.c01_V1V2	C2101_c01.V1V2.AE	CRF01_AE.TH.99.u
		gp70-BJOX002000.03.2	BJOX002000_03_2.V1V2.BC	CRF07_BC.CN.07.1-2
		gp70-CM244.ec1 V1V2	CM244_ec1.V1V2.AE	CRF01_AE.TH.90.6
		gp70-RHPA4259.7 V1V2	RHPA4259_7.V1V2.B	B.US.00.5
		gp70-TT31P.2F10.2792 V1V2	TT31P_2F10_2792.V1V2.B	B.TT.98.2
		AE.A244 V1V2 Tags/293F	A244 V1V2 Tags/293F.AE	CRF01_AE.TH.90.6
ICS	-	1086 gp120	Env.1086.C	-
	-	TV1 gp120	Env.TV1.C	-
	-	ZM96 gp120	Env.ZM96.C	-
	-	Env 92TH023	Env.92TH023.AE	-
TZM-Bl nAb	EPV**	TV1c8.2	TV1c8.2.C	C.ZA.98.6
	EPV**	Ce1086_B2	Ce1086_B2.C	C.MW.04.1-2
	EPV**	96ZM651.2	96ZM651.C	C.ZM.96.6
	EPV**	MW965.26	MW965.26.C	C.MW.93.6
	EPV**	CH0505.w4.3	CH0505.w4.3.C	C.MW.08.1-4
	EPV**	SO032_A2.8-1	SO032_A2.8-1.C	C.ZA.08.5-6
	EPV**	6644.v2.c33	6644.v2.c33.C	C.TZ.04.5-6
	EPV**	CA146 H3.3	CA146 H3.3.C	C.ZA.09.a
	EPV**	1107356.07	1107356.07.C	C.ZA.08.3

*Subtype is denoted by a capital letter; country of origin is denoted by the 2 digit International Organization for Standardization code; year isolated is denoted by 2 digits; and stage is denoted by “a” (acute, if Fiebig stage is unknown) or “1”, “2”, “3”, “4”, “5”, or “6” (acute or early chronic, where the number or range corresponds to the Fiebig stage or range of stages when known).
**EPV = Env-pseudotyped virus

Supplementary Table 2: Estimated expected probabilities of response for common HVTN 100 CD4+ T cell subsets to vaccine-matched Env antigens among vaccine recipients in the per-protocol cohorts of HVTN 100 and RV144 two weeks after the Month 6 vaccination, estimated as the average of the individual-specific estimated probabilities that the given T cell subset is positive.

T cell subset	HVTN 100 Expected probability of response to Env.ZM96.C (95% CI)	RV144 Expected probability of response to Env.92TH023.AE (95% CI)	p-value
IL2+CD40L+	68·0%, (62·2%, 73·8%)	64·4% (58·1%, 70·7%)	0·12
TNFα+IL2+CD40L+	87·8% (83·6%, 92·1%)	73·9% (68·2%, 79·6%)	<0·0001
TNFα+IL2+IL4+CD40L+	42·7% (35·6%, 49·8%)	18·8% (13·0%, 24·7%)	<0·0001
TNFα+IL2+IFNγ+CD40L+	73·0% (67·8%, 78·2%)	72·7% (67·0%, 78·5%)	0·99
TNFα+IL2+IL4+IFNγ+CD40L+	43·6% (36·3%, 50·8%)	0·0%	<0·0001

Supplementary Table 3: Mean differences, 95% CIs, and paired t-test p-values comparing BAMA responses in serum vs plasma in HVTN097 samples. Highlighted rows indicate significant differences in serum vs plasma samples.

	Log10 Differences Serum-Plasma			
**Antigen**	**Mean**	**Lower 95% CI**	**Upper 95% CI**	**p-value**
1086C_D7gp120.avi/293F	0·010	-·006	0·025	0·22
**92TH023_D11gp120**	**0·094**	**0·043**	**0·145**	**0·00075**
**96ZM651.D11gp120.avi**	**0·038**	**0·009**	**0·068**	**0·011**
**A244 D11gp120_avi**	**0·061**	**0·019**	**0·102**	**0·0055**
C.1086C_V1_V2 Tags	0·025	-·012	0·061	0·18
**Con 6 gp120/B**	**0·082**	**0·035**	**0·129**	**0·0013**
Con S gp140 CFI	0·034	-·017	0·085	0·18
**MN gp120 gDneg/293F/mon**	**0·101**	**0·056**	**0·145**	**<·0001**
**TV1c8_D11gp120.avi/293F**	**0·073**	**0·045**	**0·100**	**<·0001**
gp41	0·020	-·039	0·079	0·48
gp70-TV1.21 V1V2	0·022	-·024	0·068	0·33
gp70_B.CaseA_V1_V2	0·015	-·040	0·070	0·59
